# Targeting flexibility: a structure-based computational study revealing allosteric HIV-1 protease inhibitors

**DOI:** 10.1186/1758-2946-6-S1-P48

**Published:** 2014-03-11

**Authors:** Jens Kunze, Nickolay Todoroff, Tiago Rodrigues, Petra Schneider, Gisbert Schneider

**Affiliations:** 1 Department of Chemistry and Applied Biosciences, Swiss Federal Institute of Technology (ETH), Zurich, Switzerland

## 

We present the discovery of innovative low molecular weight inhibitors against human immunodeficiency virus 1 (HIV-1) protease. Structure-based virtual screening focused on potential allosteric surface cavities revealed these compounds [1]. To identify and prioritize such cavities we performed a molecular dynamics simulation were we concentrated on flexible and transient potential binding sites. For several time-points of the simulation we computed receptor-derived pharmacophore models in the so-called hinge region ('Exo site') and screened a large screening compound library [2]. The most potent hit shows inhibition in a non-competitive mode of action.
